# Body temperature and infection in critically ill patients on continuous kidney replacement therapy

**DOI:** 10.1186/s12882-023-03225-y

**Published:** 2023-06-07

**Authors:** Douglas W. Challener, Xiaolan Gao, Shahrzad Tehranian, Kianoush B. Kashani, John C. O’Horo

**Affiliations:** 1 Division of Infectious Diseases, Mayo Clinic, 200 First Street SW, Rochester, MN 55905 USA; 2 Division of Nephrology and Hypertension, Mayo Clinic, MN Rochester, USA; 3 Division of Pulmonary and Critical Care Medicine, Mayo Clinic, Rochester, MN USA

**Keywords:** Dialysis, Fever, Hypothermia, Sepsis, Thermoregulation

## Abstract

**Purpose:**

Continuous kidney replacement therapy (CKRT) is an increasingly common intervention for critically ill patients with kidney failure. Because CKRT affects body temperature, detecting infections in patients on CKRT is challenging. Understanding the relation between CKRT and body temperature may facilitate earlier detection of infection.

**Methods:**

We retrospectively reviewed adult patients (≥ 18 years) admitted to the intensive care unit at Mayo Clinic in Rochester, Minnesota, from December 1, 2006, through November 31, 2015, who required CKRT. We summarized central body temperatures for these patients according to the presence or absence of infection.

**Results:**

We identified 587 patients who underwent CKRT during the study period, of whom 365 had infections, and 222 did not have infections. We observed no statistically significant differences in minimum (*P* = .70), maximum (*P* = .22), or mean (*P* = .55) central body temperature for patients on CKRT with infection vs. those without infection. While not on CKRT (before CKRT initiation and after cessation), all three body temperature measurements were significantly higher in patients with infection than in those without infection (all *P* < .02).

**Conclusion:**

Body temperature is insufficient to indicate an infection in critically ill patients on CKRT. Clinicians should remain watchful for other signs, symptoms, and indications of infection in patients on CKRT because of expected high infection rates.

**Supplementary Information:**

The online version contains supplementary material available at 10.1186/s12882-023-03225-y.

## Introduction

Nearly 25% of patients admitted to intensive care units (ICUs) have acute kidney injury, associated with increased mortality rates [1]. Depending on the severity of the injury, 10% to 50% of critically ill patients with acute kidney injury require kidney replacement therapy (KRT), and approximately 2% to 4% of patients admitted to the ICU require continuous KRT (CKRT). CKRT has become the treatment of choice for such patients [2, 3], but CKRT can increase infection risk while also making detecting sepsis more challenging [4].

Body temperature is closely monitored in the ICU because alterations in body temperature are common and are associated with poor clinical outcomes, which include infection and death [5]. Com serum mon ICU interventions, such as antipyretic and intravenous fluid administration, may affect body temperature [6]. CKRT may also alter body temperature with cooling at initiation and warming at cessation [7]. However, how often fever develops in patients who undergo CKRT and have a concurrent infection is unknown.

Patients on CKRT commonly develop infections in the ICU [8, 9]. The presence of a dialysis catheter, for example, is a risk factor for infection [10]. Catheter-related bloodstream infections occur in 5% of patients on CKRT; overall infection rates are as high as 50% for those on CKRT [11, 12]. CKRT alters physiologic processes in the body, which can interfere with the clinical and biological parameters monitored for infection diagnoses. Body temperature is a particularly affected parameter [13–15]. This effect is most likely mediated through multiple mechanisms. Filtration of proinflammatory cytokines and heat loss in the extracorporeal circuit are some of the most important factors affecting body temperature [16–21]. Hypothermia occurs more commonly in patients on CKRT than in patients undergoing other modes of KRT, such as intermittent hemodialysis [22, 23].

Early detection and treatment of infection in the ICU are associated with improved patient outcomes and decreased mortality rates [24]. Patients on CKRT, however, are a specific population that may have atypical characteristics of infection. Therefore, it is essential to study commonly used markers of infection, such as body temperature, in this population. This study aims to describe the body temperature dynamics of patients with and without infection who underwent CKRT in the ICU at our institution.

## Methods

The Mayo Clinic Institutional Review Board exempted this study with a waiver for informed consent. In this historical cohort study, we retrospectively searched the electronic health records of adult patients (≥ 18 years) who required CKRT in the ICU at Mayo Clinic, Rochester, Minnesota, between December 1, 2006, and November 31, 2015. All patients in the study underwent continuous venovenous hemofiltration CKRT. Patients who declined access to their health records for research purposes were excluded from the study. Only data collected during the first ICU admission were analyzed for patients with more than 1 ICU admission with CKRT during the study period. Central body temperatures were measured via intravenous, bladder, rectal, or esophageal sensors. Patients without documented central body temperatures were also excluded from the study.

Physician reviewers determined clinical infections (D.W.C., X.G., and S.T.). The physician reviewers used a standardized protocol to assess patient health records for the presence of infection, type of infection, and estimated date of infection onset during the ICU stay (Supplemental Figure). Patients were considered to be in the infection group if the onset of infection occurred either in the five days leading to CKRT initiation or during CKRT. For patients with multiple sites of infection, we recorded all potential sources. We reviewed a random selection of approximately 10% of the abstracted health record data a second time to assess interrater reliability, measured by the Cohen κ coefficient. Central body temperature data were summarized as the median (IQR) of the minimum, maximum, mean, and SD values measured during the entire ICU stay. Body temperatures measured both before initiation and after cessation of CKRT were considered to be recorded when the patient was *off CKRT*. Conversely, body temperatures measured during CKRT were considered to be recorded while the patient was *on CKRT*. Each patient was also assessed for the presence of a single central body temperature higher than 38.0 °C and 38.3 °C, which are 2 common threshold measurements for fever. Other continuous variables were summarized as median (IQR). Categorical variables were summarized as frequency (%). All continuous data were compared with Kruskal–Wallis rank sum tests, and categorical data were compared with Fisher exact tests. Mean (95% CI) body temperature measurements recorded 24 h before initiation of CKRT (i.e., off CKRT) and 48 h after initiation (i.e., on CKRT) were plotted only for patients who had body temperature measurements documented during this period. Statistical analyses were performed with R v4.1.2 (The R Foundation) and Python 3 (Python Software Foundation) [25, 26]. *P* values less than 0.05 were considered statistically significant.

## Results

We identified 587 patients who underwent CKRT in the ICU during the study period, of whom 348 (59.3%) were men. The median (IQR) age of all patients was 62 (52–71) years, and 295 (50.3%) patients received a consultation with the infectious diseases department during their hospitalization. The median (IQR) ICU length of stay and time to CKRT after ICU admission was 10 (6–16) days and 1.4 (0.7–2.7) days, respectively. The median (IQR) duration of CKRT was 5.1 (2.9–9.3) days. The physician review determined that 365 (62.2%) patients had an infection in the ICU, with 278 (76.2%) of these infections occurring before CKRT initiation. The physician reviewers agreed 95% of the time, with a Cohen κ coefficient of 0.905 (*P* < 0.01).

A total of 381 infections occurred in 365 patients. Infections in most patients involved the pulmonary (*n* = 134, 36.7%) or gastrointestinal (*n* = 86, 23.6%) systems. Other sites of infection in these patients included skin and soft tissue (*n* = 35, 9.6%), the cardiovascular system (*n* = 64, 17.5%), the central nervous system (*n* = 3, 0.8%), the urinary system (*n* = 25, 6.8%), and other sites (*n* = 17, 4.7%). No infection source was identified for 17 patients, but the physician reviewers concluded that an infection was most likely present. Therefore, these patients were included in the *with infection* group.

**Fig. 1:**
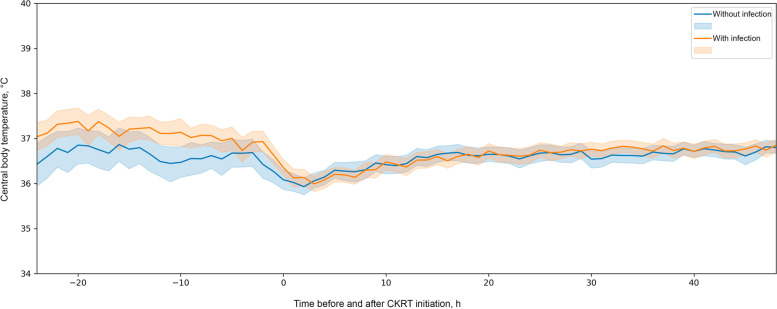
Central Body Temperature Measurement in Patients on Continuous Kidney Replacement Theraphy (CKRT) in the Intensive Care Unit. Mean body temperature measurements recorded 24 h before initiation of CRKT (off CKRT) and 48 h after initiation (on CKRT) were plotted only for patients who had measurements documented during this time period. Solid lines indicate mean temperature, and shaded regions indicate 95% CI

The body temperatures of patients with and without infections while on and off CKRT are reported in the Table. During CKRT, the minimum (*P* = 0.70), maximum (*P* = 0.22), and mean (*P* = 0.55) central body temperatures did not significantly differ between patients with or without infections. However, the body temperature SD was larger for patients with infection (0.64 °C) than for those without infection (0.55 °C), which indicates that body temperature variability was significantly greater for patients with infection (*P* = 0.01). When the patients were off CKRT, the median (IQR) minimum (36.0 [34.7–36.7] °C vs. 35.8 [34.3–36.4] °C), maximum (37.9 [37.2–38.7] °C gvs 37.5 [37.0–38.2] °C), and mean (36.9 [36.3–37.5] °C vs. 36.7 [36.0–37.1] °C) body temperatures were all significantly higher in patients with infection than in those without infection (all *P* < 0.02). Temporal changes in mean body temperatures 24 h before and 48 h after initiating CKRT for patients with and without infection are depicted in Fig. 1.

The number of patients with a body temperature measurement higher than 38.0 and 38.3 °C is reported in Table 1. Of the patients with infection, 152 (46.1%) had a body temperature higher than 38.0 °C while off CKRT. While on CKRT, this number decreased to 124 (34.0%). The number of patients with a recorded body temperature higher than 38.3 °C was significantly higher in patients with infection than in those without infection, both on CKRT (*P* = 0.03) and off CKRT (*P* < 0.001).

**Table 1:** Summary of Patient body temperatures in the intensive care unit according to the presence of infection and use of CKRT^a^

CKRT status	Body temperature measurement, °C	Total	Without infection	With infection	*P*
On CKRT		**(*N* = 587)**	**(*n* = 222)**	**(*n* = 365)**	
	Minimum	35.1 (33.9–35.9)	35.1 (33.6–35.8)	35.1 (34.1–35.9)	.70^b^
	Maximum	37.7 (37.1–38.3)	37.7 (37.2–38.1)	37.7 (37.1–38.3)	.22^b^
	Mean	36.5 (36.2–36.9)	36.5 (36.2–36.9)	36.5 (36.1–37.0)	.55^b^
	SD	0.60 (0.42–0.83)	0.55 (0.40–0.76)	0.64 (0.45–0.84)	.01^b^
	> 38.0	182 (31.0)	58 (26.1)	124 (34.0)	.05^c^
	> 38.3	129 (22.0)	38 (17.1)	91 (24.9)	.03^c^
Off CKRT		**(*n* = 536)**^**d**^	**(*n* = 206)**^**d**^	**(*n* = 330)**^**d**^	
	Minimum	35.9 (34.6–36.6)	35.8 (34.3–36.4)	36.0 (34.7–36.7)	.02^b^
	Maximum	37.8 (37.1–38.6)	37.5 (37.0–38.2)	37.9 (37.2–38.7)	< .001^b^
	Mean	36.9 (36.2–37.4)	36.7 (36.0–37.1)	36.9 (36.3–37.5)	< .001^b^
	SD	0.52 (0.32–0.82)	0.53 (0.31–0.82)	0.52 (0.32–0.82)	.95^b^
	> 38.0	211 (39.4)	59 (28.6)	152 (46.1)	< .001^c^
	> 38.3	169 (31.5)	47 (22.8)	122 (37.0)	< .001^c^

*Abbreviation*: *CKRT* continuous kidney replacement therapy

^a^Continuous data (minimum, maximum, mean, and SD) are summarized as median (IQR), and categorical data (> 38.0 °C and > 38.3 °C) are summarized as No. (%) of patients

^b^*P* value determined with Kruskal–Wallis rank sum test

^c^*P* value determined with Fisher exact test for count data

^d^Some patients did not have central body temperatures measured while off CKRT

## Discussion

Body temperature differences between patients with and without infection appeared to be minimized in patients on CKRT. SD was the only body temperature measurement that differed between patients with and without infection during CKRT. However, the body temperature SD was only approximately 0.1 °C higher in those with infection than in those without infection while on CKRT. Therefore, the clinical relevance of SD to the detection of infection-related fever is limited. Nevertheless, the apparent differences in minimum, maximum, and mean body temperature measurements between patients with and without infection while off CKRT suggest that measuring body temperature before initiating CKRT may be most useful for detecting infection. When infection is suspected in patients on CKRT, a brief disruption may unmask changes in body temperature.

Defining a threshold of 38.3 °C allowed us to distinguish between patients with and without infection, even while on CKRT. However, only 34.8% of patients with infection met this criterion while on CKRT. This reinforces the value of thoughtful clinical evaluation and use of signs and symptoms other than body temperature when assessing for the presence of infection during CKRT.

To our knowledge, this study is the first to evaluate body temperature dynamics in patients with infection while on CKRT. Additionally, the abstraction of infection status by expert physician reviewers allowed for curating high-quality outcome data. Therefore, this study provides a reliable estimate of the potential effects of CKRT on central body temperature. Despite these strengths, this study also has several limitations that must be addressed in follow-up investigations. Because this was a historical cohort study, it was subject to biases. Determining the presence of an infection post hoc is challenging. The determination of infection relied heavily upon the evaluation of care team documentation. If a patient had an infection at any time in the five days before CKRT or while on CKRT, they were included in the infection group, which does not account for patients who may have suffered from a transient infection that resolved quickly. Although measures were taken to provide a strong case definition of infection with high interrater reliability, the generalizability of this definition in other contexts is unclear. In addition, all patients in the study underwent continuous venovenous hemofiltration. Because other types of CKRT were not analyzed, the applicability of our findings to other centers may be limited. Lastly, there are a multitude of other factors besides fever that may impact a patient's body temperature that were not examined in this study. Future studies should focus on the prospective evaluation of body temperature dynamics and include other factors that may affect body temperature (e.g., pyrolytics, cooling devices, presence of pulmonary emboli).

## Conclusions

As CKRT continues to be used with increasing frequency in ICUs, knowledge about how its use may affect markers of infection is helpful for its interpretation by treating clinicians. Our findings indicate that body temperature is insufficient as a sole indicator of the presence of infection in critically ill patients on CKRT. Therefore, clinicians should remain vigilant for infection in patients on CKRT because of its association with high infection rates. All available clinical data should be used, and cultures of relevant sites should be obtained if an infection is suspected.

## Supplementary Information

**Additional file 1.** 

## Data Availability

All relevant data supporting the findings of this study are reported in the article.

## Abbreviations

CKRT: Continuous kidney replacement therapy
ICU: Intensive care unit
KRT: Kidney replacement therapy
