# Supplementary figures and images for: Body temperature and infection in critically ill patients on continuous kidney replacement therapy
Source: BMC Nephrol. 2023 Jun 7;24:161. doi: 10.1186/s12882-023-03225-y (PMC10245466; doi:10.1186/s12882-023-03225-y)

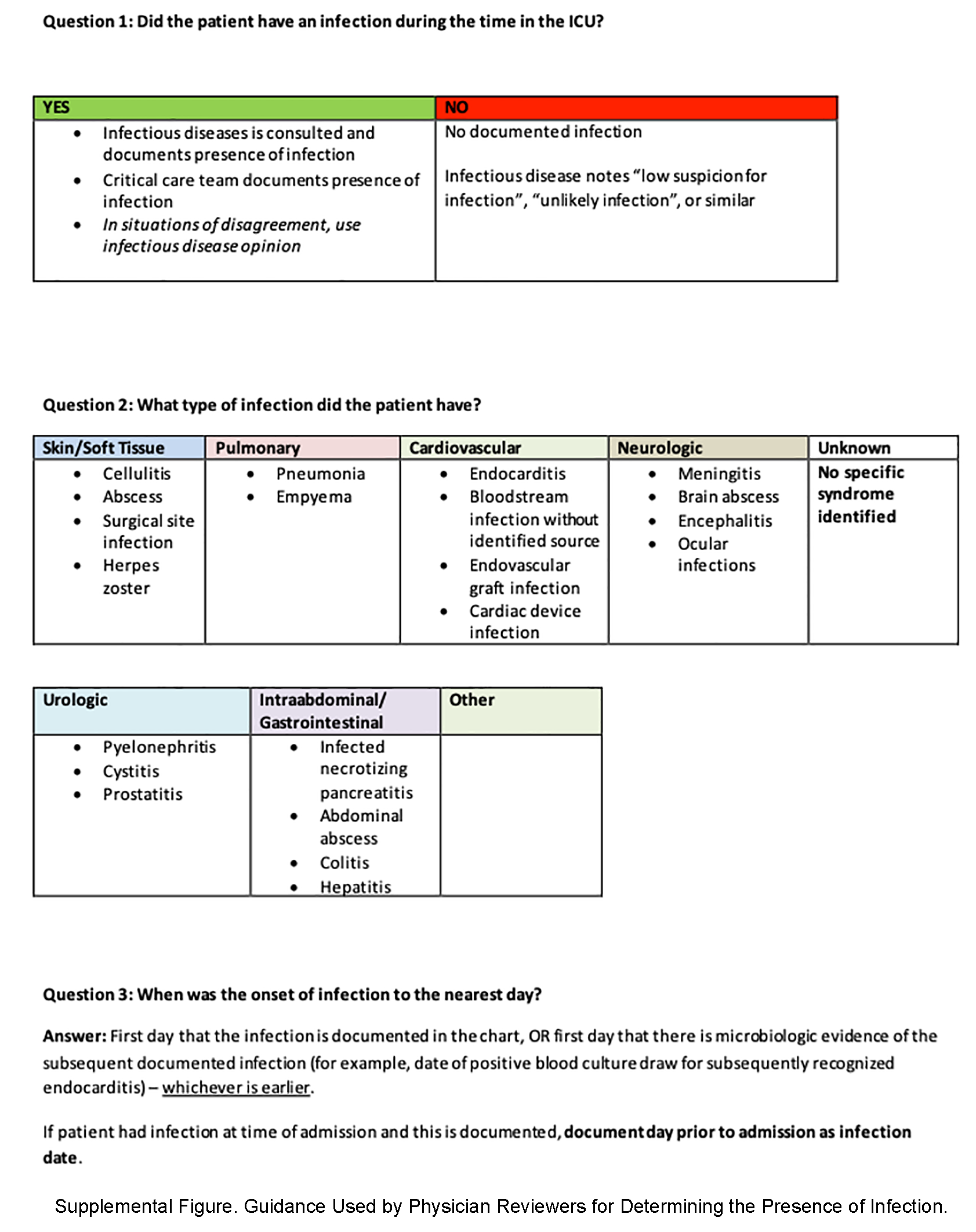

Supplement: Supplementary file 1 — Additional file 1. [file 12882_2023_3225_MOESM1_ESM.tif]
